# Cardiac hypertrophy and insulin therapy in a pre-term newborn: is there a relationship?

**DOI:** 10.1186/s13052-022-01216-7

**Published:** 2022-02-08

**Authors:** Guglielmo Salvatori, Giulia Brindisi, Mario Colantonio, Anna Maria Zicari

**Affiliations:** 1 Neonatal Intensive Care Unit and Human Milk Bank, Department of Neonatology, Bambino Gesù Children’s Hospital, IRCSS, Rome, Italy; 2 Pediatrics Department, Umberto I Hospital, Sapienza University, Rome, Italy; 3 Department of Maternal Infantile and Urological Sciences, Division of Pediatric Allergology and Immunology, Sapienza University of Rome, Rome, Italy; 4 Department of Neonatology, S. Camillo Forlanini Hospital, Rome, Italy

**Keywords:** Hyperinsulinemia, Hypertrophic cardiomyopathy, Hyperglycemia, Parenteral nutrition (PN), extremely low birth weight (ELBW)

## Abstract

**Background:**

Hypertrophic cardiomyopathy (HCM) in newborns is a rare condition with heterogeneous etiologies. While the relationship between hyperinsulinism and cardiac hypertrophy (CH) is known, hyperinsulinism has not been reported as cause of HCM.

**Case presentation:**

We report the case of cardiac hypertrophy (CH) in an Extremely Low Birth Weight (ELBW) infant; this patient underwent insulin therapy after the onset of persistent hyperglycemia due to parenteral nutrition (PN), supporting the hypothesis of a role of iatrogenic hyperinsulinemia in the development of HCM.

**Conclusions:**

The present case underlines the importance of a close cardiological follow-up in infants undergoing insulin infusion for an alteration in the glucose metabolism.

## Introduction

Hypertrophic cardiomyopathy (HCM) in newborns is a rare pathological condition in which disruption of the myocardial structures creates a thickening of the heart muscle [1]. CH is defined by echocardiographic diastolic septal thickness or diastolic left ventricular wall thickness equal to ≥2 standard deviations above the mean (Z-score ≥ 1.96; corrected for age, sex, and body size) [2–4]. The etiology of HCM is extremely heterogeneous, including malformation syndromes, inborn errors of metabolism, neuromuscular disorders, and in the majority of the cases mutations in cardiac sarcomere protein genes [5]. While hyperinsulinism has not been reported, the relationship between hyperinsulinism and cardiac hypertrophy (CH) is well known. In fact, hyperinsulinism is mostly linked to CH rather than HCM, which requires histological and functional disaggregation’s criteria. The cause of CH is particularly important to understand, especially for its implications in clinical practice and for its prognostic and/or therapeutic consequences [1].

In newborns and infants hyperinsulinism can have many different causes: congenital hyperinsulinism (transient and persistent), maternal diabetes, insulin resistance syndromes, hyperinsulinism syndrome-related (such as Becwith-Wiedemann, Costello, Noonan and Leopard syndrome) and iatrogenic hyperinsulinism (excessive infusion of insulin) [1].

In neonates with congenital hyperinsulinism, fetal hyperinsulinemia increases the storage of glucose and lipids with a consequent hyperplasia and hypertrophy of myocardial cells. Indeed, septal thickness gradually decreases until it becomes normal within the first few months of life, usually without complications and requiring beta-blockers only in rare cases [6]. Similarly, in maternal diabetes, hyperglycemia can produce fetal hyperinsulinemia that can persist until the neonatal period. Hyperglycemia is a well known teratogen factor [7, 8] that can result in a variety of cardiac defects [9–12].

Conversely, the persistent congenital hyperinsulinism is due to a focal or diffuse overproduction of insulin originated by the pancreas in relation to various genetic disorders [6].

Even though epidemiologic and prospective studies have shown that glycosylated hemoglobin A1c level in the 6 months before conception and during the first trimester of pregnancy correlates with the increased incidence of major malformations (such as neural tube, cardiac defects and spontaneous abortions), the specific effects of hyperglycemia are still unclear [13, 14].

Nevertheless gestational diabetes acts with a teratogen effect on the embryo already from the first weeks of gestation. Consequences are primary defects of cardiogenesis and asymmetric hypertrophic cardiomyopathy with a thickening of the interventricular septum and lower posterior ventricular wall [15]. Infants of diabetic mothers show an incidence of congenital cardiac disease of about 3.6%, compared to 0.8% in the general population [16]. In particular among the 30% of infants without congenital cardiac diseases born from diabetic mothers, the echocardiographic exam presents an interventricular septum and ventricular walls hypertrophy with a ratio from interventricular septal / left posterior ventricle wall higher than 1,3 [17].

There are also cases of hyperinsulinemia secondary to an excessive infusion of insulin, as in the case report presented. A multicenter case-control study, conducted by Bearsall et al., showed that insulin infusion administered to hyperglycemic VLBW infants in the first weeks of life resulted in a significant gain of glucose and a total energy intake. However, an increased risk of hypoglycemia was reported, without a significant reduction of mortality and morbidity [18].

Insulin has the role of anabolic hormone and acts as cardiac growth factor, promoting cardiac hypertrophy due to the interaction with its receptors on heart muscle cells. Therefore, CH related to a hyperinsulinemic condition is characterized by an exuberant growth of single myocardiocytes, affecting the heart as a whole [1]. In the majority of cases, cardiac hypertrophy due to a hyperinsulinemic condition is asymptomatic and reversible after the normalization of insulin levels [19–22].

For the differential diagnosis of hyperinsulinism in newborns, see Table 1 below.

**Table 1:** Differential diagnosis of hyperinsulinism in newborns [6–10]

Disease	Hyperinsulinism type	Hyperinsulinism trigger cause/etiology
Transient Congenital hyperinsulinism	Transient	- Maternal Stress - Asphyxia - Intrauterine growth restriction
Persistent Congenital hyperinsulinism (also named persistent hyperinsulinemic hypoglycemia of infancy)	Persistent	Genetic disorders with mutations in the Kir6.2 (KCNJ11 gene, omim #600937) and SUR1 (ABCC8 gene, omim #600509) that cause a focal or diffuse overproduction of insulin in the pancreas
Maternal Diabetes	Transient	Gestational diabetes
Hyperinsulinism drug-related	Transient	Excessive infusion of insulin
Congenital disorders of glycosylation	Persistent	Autosomal recessive disorders characterized by defective biosynthesis of lipid-linked oligosaccharides or compromised processing of protein-bound oligo- saccharides
Becwith-Wiedemann syndrome	Persistent	Complex multigenic hyperinsulinism syndrome caused by dysregulation of imprinted growth regulatory genes within the chromosome 11p15 region, including increased activity of the IGF-2 gene in several tissues
Costello syndrome	Persistent	Congenital hyperinsulinism syndrome caused by mu- tations in the HRAS proto-oncogene

## Case presentation

We describe the case of a ELBW infant (25 +  1 weeks of gestational age, birth weight 880 g, length 33 cm, cranial circumference 24 cm), born from a caesarean section due to a cervical insufficiency. The mother was a multiparous 27-year-old woman who had shown a shortening of the uterine cervix in the last month of pregnancy. For this condition, she was undergoing isoxsuprine hydrochloride therapy to reduce uterine contractions and the possible risk of a premature birth.

Few hours before delivery, an intravenous glucocorticoid bolus was administered. The APGAR score was six at 1 min of life; then the newborn underwent orotracheal intubation due to the presence of respiratory failure.

Surfactant was administered, followed by 96 h of mechanically ventilation. Following extubation the newborn was supported with nasal continuous positive air pressure (n-CPAP) and oxygen for 7 days to maintain oxygen saturation between 90 and 95%. Due to jaundice, phototherapy was administered until the fourth day of life.

Because of the presence of a hemodynamically significant patent ductus arteriosus, ibuprofen was started (10 mg/Kg, 5 mg/Kg, 5 Kg/mg to 24 h apart) and eritroprotein (250 units 3 times /week) for the anemia of the prematurity. No alterations of procalcitonin (PCT) and C-reactive protein (CRP) were detected excluding the diagnosis of sepsis. Feeding intolerance (gastric residuals, abdominal distension) required the starting of full parenteral nutrition. After 3 days of therapies, persistent hyperglycemia arose (217 mg/dL).

Newborn’s glucose intake was 11.5 mg/kg/min, subsequently reduced to 10 mg/kg/min, before starting a continuous insulin infusion. Insulin infusion was administered for 10 days and its rate was adjusted between 0.1 UI/Kg/h to 0.01 UI/Kg/h to obtain the stabilization of blood glucose levels, reached after only 6 days from the beginning of insulin treatment.

On the 15th day of life, a systolic ejection murmur in the mesocardium (2/6) was detected, triggering an echocardiographic exam which showed hypertrophic cardiomyopathy with thickness of the septum and reduction of ventricular cavity, see Table 2 below. Other findings were: reduction of velocity in ventricular outflow; right trabecular hypertrophy; mild mitral valve insufficiency due to lack of coaptation of the mitral valve leaflets’ secondary to rapidly established hypertrophy with a ventricular cavity that has suddenly become too small.

**Table 2:** Thickness of the septum and diameter of the ventricular cavity

Thickness:	mm	Z score
**• Interventricular septum (diastolic)**	5.5	+ 2.52
**• Interventricular septum (systolic)**	6.4	+ 2.49
**• Free wall - posterior left ventricular wall (diastolic)**	4.7	+ 3.54
**• Free wall -posterior left ventricular wall (systolic)**	0.65	+ 2.38
**Diameter:**		
**• Left ventricular end diastolic**	16.5	+ 1.35
**• Left ventriclular end systolic**	0.87	- 0.64

The infant developed also some episodes of desaturation during n-CPAP and a doxapram hydrochloride infusion was started (0.5 mg/kg/h).

Cardiac ultrasonography showed: on the 17th day of life, electrocardiogram (ECG) presented an elevated ST segment, particularly in the left precordial leads. Subsequently insulin infusion was suspended.

After 10 days from insulin suspension, the echocardiographic ultrasound exam showed a progressive reduction of free left wall septum and of trabecular hypertrophy.

After 40 days of life, the ECG showed a normal tracing and the echocardiographic ultrasound revealed a further improvement in cardiac hypertrophy.

At the time of discharge, the baby did not present retinopathy of prematurity, hypertension, renal failure, but outcomes of bronchodysplasia. During the follow-up and up to age of 36 months, was in good clinical condition.

## Discussion

The goal of nutrition for preterm infants should be to achieve a postnatal growth rate approximately equal to the rate of a normal fetus with the same gestational age. Unfortunately, most preterm infants, especially ELBW, are not fed sufficient amounts of nutrients to reach normal fetal growth rates. The resulting extrauterine growth retardation is a significant problem that could result in short stature, organ growth failure, neuronal deficits with behavioral problems, and poor cognitive outcomes [23, 24]. Therefore it is very important to provide nutrients immediately after birth with parenteral nutrition (PN). However, in the first weeks of life, ELBW infants might present hyperglycemia in 40–80% of cases [25]. In ELBW infants hyperglycemia increases the risk of early death, intraventricular hemorrhage and extends the length of hospitalization, which suggests that prevention and treatment of hyperglycemia may improve the outcomes for these infants [26, 27].

Potential causes of hyperglycemia include sepsis, necrotizing enterocolitis, surgical treatments, infusion of vasoactive drugs and steroids, insulin resistance and/or relative insulin deficiency and high glucose intake, as in the case described above. The standard approach to the management of hyperglycemia in infants involves the use of glucose restriction and/or continuous insulin infusion to achieve a normal level of glycaemia, while maintaining an adequate caloric intake [28, 29].

This treatment can prevent osmotic diuresis, dehydration and electrolyte imbalance. However, insulin infusion is not free from serious complications, including hypoglycemia, that remains an important and risky possible consequence [30].

To the best of our knowledge, the case we describe here is the setting of the first report of an ELBW newborn who showed a transient cardiac hypertrophy in association to iatrogenic hyperinsulinemia. A possible correlation is supported by the close temporal relationship between cardiac hypertrophy and insulin infusion, and its resolution after suspension of insulin therapy. Other cases of relationship between CH and hyperinsulinism are described in the literature, mainly in the setting of congenital hyperninsulinism or maternal diabetes [16, 31–33].

As for the mechanism of this association, Kruger M. et al. explained the association between cardiac hypertrophy and hyperninsulinism in diabetic rats, showing that high fetal insulin decreases the expression of the N2B titin protein isoform in cardiac cells [34]. Titin protein is the main mechanism for adjusting passive myocardial stiffness in perinatal heart development and in chronic heart disease. Insulin controls the cardiac titin-isoform pattern by activating the phosphoinositol-3-kinase pathway [34]. Therefore, insulin signaling regulates both titin-isoform composition and titin phosphorylation in embryonic cardiomyocytes and could contribute to an altered diastolic function in diabetic cardiomyopathy [34]. Other authors suggested that chronic hyperinsulinemia in rats might increase body weight, tibia length, heart weight and left ventricular mass thanks to the anabolic function of this hormone [35–38].

A recent review by Paauw N.D. et al., emphasizes the role of insulin as a cardiac growth factor in hyperinsulinemic infants with CH [1]. Indeed, the heart is an important target for the anabolic effect of insulin, which interacts with its receptors on cardiomyocytes, thus promoting CH [39, 40]. CH is found in many different hyperinsulinemic conditions, supporting the fact that CH develops regardless of the underlying cause of the high insulin levels [1].

In this perspective, we speculate that the pathophysiology underlying CH in the described infant might be similar to the one assessed in hypertrophic cardiomyopathy of diabetic mothers’ infants or in congenital hyperinsulinism [22, 33, 41, 42].

This is the first case described in the literature of an ELBW, in whom a transient cardiac isolated condition, not associated with other pathologies, was associated with iatrogenic hyperinsulinemia. This underlines the importance of a close follow-up in infants under insulin treatment, consequent to an altered glucose metabolism during PN.

In conclusion, hyperinsulinism is linked to CH in many different hyperinsulinemic diseases and should be listed in the differential diagnosis of myocardial hypertrophy. Further studies are warranted to better define the potential consequences of the prolonged use of insulin therapy on the heart, and to clarify the cost-benefit relationship of this therapy in ELBW infants undergoing PN, for a period of their life.

## Abbreviations

n-CPAP: Nasal continuous positive air pressure
PN: Parenteral Nutrition
ELBW: Extremely Low Birth Weight
VLBW: Very Low Birth Weight
ECG: Electrocardiogram
ROP: Retinopathy of prematurity
